# OWL2 benchmarking for the evaluation of knowledge based systems

**DOI:** 10.1371/journal.pone.0179578

**Published:** 2017-06-20

**Authors:** Sher Afgun Khan, Muhammad Abdul Qadir, Muhammad Azeem Abbas, Muhammad Tanvir Afzal

**Affiliations:** Centre for Distributed and Semantic Computing, Capital University of Science and Technology, Islamabad, Pakistan; Ege Universitesi Muhendislik Fakultesi, TURKEY

## Abstract

OWL2 semantics are becoming increasingly popular for the real domain applications like Gene engineering and health MIS. The present work identifies the research gap that negligible attention has been paid to the performance evaluation of Knowledge Base Systems (KBS) using OWL2 semantics. To fulfil this identified research gap, an OWL2 benchmark for the evaluation of KBS is proposed. The proposed benchmark addresses the foundational blocks of an ontology benchmark i.e. data schema, workload and performance metrics. The proposed benchmark is tested on memory based, file based, relational database and graph based KBS for performance and scalability measures. The results show that the proposed benchmark is able to evaluate the behaviour of different state of the art KBS on OWL2 semantics. On the basis of the results, the end users (i.e. domain expert) would be able to select a suitable KBS appropriate for his domain.

## Introduction

Ontologies are extensively used in the scientific domains like Gene engineering and life critical systems. These domains carry their own complexity based on schema, number of axioms and the OWL semantics. For instance, a memory based KBS might be a suitable choice to represent the Agriculture (fao.org) ontology. In contrast, Gene (geneontology.org) ontology might require a persistent storage model because of its high schematic complexity that exists in the form of multiple parents. In the present work, KBS refers to ontology based KBS. These systems differ from each other on the basis of their storage model, schema and expressiveness [1]. Storage can be memory based, file based, database, or graph based. Moreover, expressiveness can be represented in RDF, RDFS, OWL or OWL2.

Choosing an appropriate KBS for OWL2 ontologies is deemed to be an important and critical task that helps the end user to select the most suitable system for their domain. This selection requires an evaluation of the existing KBS. The present work addresses the evaluation of the existing KBS using the proposed evaluation benchmark for OWL2 ontologies. The past decade has witnessed a number of evaluation benchmarks for the KBS such as Lehigh University Benchmark(LUBM) (1), University Ontology Benchmark (UOBM) [2], Berlin SPARQL Benchmark(BSBM) [3], Dbpedia benchmark [4], SP2B [5], OntoBench [6], etc. However, current state of the art benchmarks lacks OWL2 evaluation and focuses only on the OWL semantics.

The proposed evaluation benchmark focuses on OWL2 semantics because it is the need of the current real applications to model complex domains [7]. These domain requirements are fulfilled by additional expressiveness (new syntaxes, addition of profiles, provision of type separations and language enhancements) of OWL2. The present work addresses the building blocks of a standard evaluation benchmark i.e. data schema, workload and performance metrics. The details of these building blocks are provided in the next section.

The methodology of the present work comprises of analysis of the existing benchmarks, construction of the data schema and workload (i.e. data generator and query set) for OWL2 semantics. The performance matrices comprise of dataset load time, query response time and scalability of KBS. As a case study, the university ontology [2] is used for the construction of proposed benchmark named as OWL2 Ontology Evaluation Benchmark (OEB2). Finally, an evaluation of seven well known KBS on three different sized datasets against the constructed queries is performed to demonstrate their behaviour on the OWL2 semantics. The results concludes that all of the KBS (Jena SDB [jena.apache.org/documentation/sdb/], Sesame [rdf4j.org], Blazegraph [blazegraph.com], Owl2ToRDB [8], OntRel [9]) exhibit similar behavior on concept or property based queries but the performance drastically varies on complex and property pattern based queries for different sized datasets. On the basis of these findings, end user (i.e. domain expert) would be able to choose a suitable KBS.

The structure of the paper is organized as follows. Section 2 describes the literature review and analysis of the existing benchmarks. Section 3 describes the proposed evaluation benchmark and its components. Section 4 evaluates the proposed benchmark. Section 5 reports the evaluation of the KBS using proposed benchmark. Section 6 describes the conclusion and future work.

## Literature review and analysis of the existing benchmarks

This section describes, reviews and evaluates elements of the existing ontology evaluation benchmarks to conclude the research gap.

### Ontology benchmark elements

State of the art has classified the evaluation benchmark elements as data schema, workload and performance metrics [1], [2], [10]. These elements are described as follows:

#### Data schema

It describes the structure of the data, usage of ontology constructs (hereafter, ontology construct refers to both OWL and OWL2) and relationship between the classes and properties. The structural complexity of the data schema and the ontology semantics are important factors in the benchmark performance. These factors provide basis for the proposed evaluation criteria, which comprises of two elements. a) Examining the data schemas of the existing benchmarks for the OWL2 semantics. The methodology for this activity is carried out by comparing the OWL2 semantics used in any data schemas with the standard OWL2 reference list [11]. b) The complexity of the data schemas of the existing benchmarks is determined by computing the edge to node ratio (EnR).

#### Workload

It comprises of dataset generation process and a set of queries. Dataset generation process of a benchmark generates different size datasets. Later, the set of queries are executed over the generated datasets to capture the behaviors of the KBSs. The evaluation of the dataset generation process is carried out by inspecting OWL2 semantics in the source code of the existing benchmarks. Moreover, the evaluation of the query sets used by the existing benchmarks is performed by evaluating the usage of OWL2 constructs. This evaluation of the query set focuses on common characteristics of the benchmark queries i.e. input size, selectivity, complexity and hierarchal inference [1], [2]. The methodology for the evaluation of benchmark query sets comprises of enlisting all the distinct ontology constructs (directly or indirectly) associated with the classes and properties used in the benchmark queries.

#### Performance metrics

It describes the performance of the ontology systems in a quantitative manner. The common performance metrics include data load time, query response time and scalability (different sized datasets).

### Ontology evaluation benchmarks

Evaluation of ontology benchmarks is an important and challenging task to check the appropriateness (efficiency and scalability) of the KBSs in different domains. There are some well-known evaluation benchmarks available for the evaluation of KBSs. LUBM is considered to be the most influencing benchmark to check the capabilities of the KBSs [1]. This benchmark [1] uses a flexible ABox dataset generation tool named UBA to generate datasets. LUBM uses fourteen queries to evaluate the performance of the KBS for data loading, repository size, query response time and combined complexity. Lehigh BibTeX Benchmark (LBBM) [10]generates the synthetic dataset on the basis of relevant extracted properties from the real domain documents.

The Berlin SPARQL Benchmark (BSBM) [3] was developed for comparing the performance between native RDF stores and systems featuring SPARQL-to-SQL rewriters. BSBM [3] has adopted an e-commerce application as their case study and mainly addressed the dataset generation process. University Ontology Benchmark (UOBM) [2] extends LUBM [1] in terms of inference and scalability. UOBM covers most of the OWL Lite and OWL DL constructs in their data schema. Major contributions of the UOBM are generation of the benchmark ontology using OWL Lite and OWL DL inference, construction of a single connected RDF graph using property connects and evaluation of several popular KBSs for inference and scalability.

OntoDBench [12] evaluates the scalability and query performance of the relational storage systems under different storage representations (vertical, horizontal and binary). The benchmark [12] addresses the storage models and query rewriting modules to perform evaluation of real world characteristics in LUBM datasets.

OntoBench [6] uses the benchmark ontologies with OWL2 coverage for testing, reviewing, and comparing the ontology visualization tools. It provides testing of an ontology tool in terms of ontology supported features and OWL2 semantics. An end user can select the elements from the ontology features like OWL Lite, OWL DL, OWL2 EL, OWL2 QL and OWL2 RL in OntoBench. Afterward, the benchmark (OntoBench) will generate the ontology version based on the user selected elements. The benchmark contributes in addressing the inflexibility and overhead issue of the static benchmark ontology. However, OntoBench supports testing of the TBox only whilst, ABox testing is not provided. Under the benchmark evaluation, OntoBench only addresses the data schema and the testing of ontology visualization tools e.g. WebVOWL. The Norwegian Petroleum Directorate (NPD) benchmark evaluates the ontology based data access systems (OBDA) using the real world dataset from the oil industry [13]. The benchmark proposed by Butt et al. [14] consists of Barton library dataset, six benchmark queries, test cases based upon CRUD operations and evaluation metrics such as resource utilization, success ratio and cumulative query performance. The benchmark is tailored to evaluate the performance and scalability of the semantic web databases. The Dbpedia benchmark [4] is a SPARQL benchmark, which consists of Dbpedia ontology, benchmark queries, SPARQL endpoint and RDF synthetic dataset generator. The benchmark queries are a selected set of user queries posed over the Dbpedia Knowledge base. The benchmark uses the query-log mining, clustering and SPARQL feature analysis to compare the RDF triple stores.

In recent years, much work has been done in the area of RDF. There is long list of RDF triplestore benchmarking available at (w3.org/wiki/RdfStoreBenchmarking). Most of the benchmarks characterize real RDF datasets, set of RDF queries and synthetic dataset generator. These benchmarks in general are tailored for the RDF stores evaluations, which does not fall in the scope of the present paper.

The existing benchmarks evaluate KBSs on OWL semantics in data schema, dataset generator and workloads. OWL2 semantics are not covered by the benchmarks except the OntoBench [6] in data schema, which generates ontologies with already defined structure alongwith the options to select OWL and OWL 2 elements. However, OntoBench [6] does not address the dataset generator and workloads because it is a benchmark for ontology generation containing OWL2 constructs. The complexity of the data schemas provided with the benchmarks is also weak [6]. Edge to Node Ratio (EnR) explains the complexity of the structure. Table 1 presents the computed EnR of the UOBM data schema, vehicle ontology [8] and OntoBench. Among OWL constructs in UOBM (refers to Table 1), the usage of *SubClassOf* relationship is very high (i.e. 0.75 for OWL-DL and 1.36 for OWL-Lite ontology) as compared to other constructs. Whereas, the EnR of OWL constructs in OntoBench is appropriate from the OWL2 coverage point of view. In UOBM, the overall EnR of OWL-DL and OWL-Lite is 2.567 and 3.292 respectively. The EnR becomes very low (0.309 and 0.487) when the OWL constructs are confiscated. This indicates that the small sized clusters of instances and classes are sparsely connected. The analysis reveals the fact that 97 classes (nodes) in OWL-DL and 89 classes of OWL-Lite are not connected with any object properties. From this point, a conclusion can be drawn that data schema of UOBM is a simple structure. While, the structure of the vehicle ontology [8] is more complex and uses more OWL constructs. OntoBench [6] exhibits the coverage of all OWL constructs. A similar method of explaining the complexity of ontology structure for computing the association or similarity between concepts through OWL constructs was presented by Wei Gao et al. [15], [16].

**Table 1:** OWL constructs usage in the benchmark and non-benchmark ontology.

OWL Constructs	University Ontology				Vehicle ontology		OntoBench Ontology	
	OWL-DL ontology		OWL-Lite ontology					
	NoE	EnR	NoE	EnR	NoE	EnR	NoE	EnR
Subclassof	85	0.75	154	1.36	52	2.74	23	0.24
Domain	27	0.24	27	0.24	17	0.89	39	0.41
Range	25	0.22	43	0.38	18	0.95	40	0.42
Subproperty	9	0.08	29	0.26	1	0.05	1	0.01
Equivalent class	22	0.19	0	0.00	2	0.11	6	0.06
IntersectionOf	20	0.18	20	0.18	0	0.00	2	0.02
SomeValuesFrom	36	0.32	22	0.19	3	0.16	11	0.12
Allvaluesfrom	6	0.05	2	0.02	5	0.26	5	0.05
ComplementOf	4	0.04	0	0.00	0	0.00	1	0.01
Unionof	1	0.01	0	0.00	8	0.42	3	0.03
Inverseof	4	0.04	5	0.04	9	0.47	1	0.01
Disjointwith	1	0.01	0	0.00	13	0.68	2	0.02
Equivalent property	1	0.01	1	0.01	1	0.05	6	0.06
Functional object property	1	0.01	1	0.01	12	0.63	1	0.01
Inverse functional object property	1	0.01	1	0.01	3	0.16	1	0.01
Transitive property	2	0.02	2	0.02	2	0.11	1	0.01
Symmetric property	2	0.02	2	0.02	1	0.05	1	0.01
Data property domain	6	0.05	6	0.05	24	1.26	57	0.60
Data property range	2	0.02	2	0.02	26	1.37	56	0.59

Object property characteristics are important for building up semantic associations among ontology classes, but UOBM uses only four property (out of nine) characteristics: functional, inverse functional, transitive and symmetric property. This shows the lack of semantics in the benchmark data schema. Similarly, the dataset generator uses limited number of class and property assertions i.e. 44.44% property axioms, 28.32% classes, 60% object properties and 66% data properties in its dataset.

Analysis in Table 2, shows that most of the benchmarks [3], [4], [9] compared with the performance of RDF stores focuses on the SPARQL benchmarking. Whilst, the benchmarks [1], [2], [13] have a commonality of ontological benchmark features. The OntoBench [6] focuses on ontologies generation with OWL2 constructs but lacks the KBS evaluation.

**Table 2:** Evaluation benchmarks and their KBS.

Evaluation benchmark	Ontology expressiveness	Evaluated ontology storage systems(KBS)	OWL constructs	
			Benchmark ontology	Benchmark Dataset
LUBM [1]	SROIN(D), OWL Lite	File based, Memory based, RDMS, RDF store	Limited use of OWL constructs	Limited use of OWL constructs
BSBM [3]	RDFS	RDMS, RDF store	-	-
UOBM [2]	SHIN(D), OWL Lite, OWL DL	File based, Memory based, RDMS, RDF store	- OWL Lite and OWL-DL complete - No support for OWL2	Limited use of owl constructs.
OntoDBench [12]	SROIN(D), OWL Lite	RDMS with three database representation	- Limited use of OWL constructs - No support for OWL2	Limited use of OWL constructs
Dbpedia [4]	ALCHF(D)	RDF store	- Limited use of RDFS /OWL constructs - No support for OWL2	-
Butt,2014 [14]	RDFS	RDF store	-	-
OntoBench(17)	SROIQ(D)	Ontology visualization tools	-Support OWL & OWL2 constructs	- Lack of support for ABox - No dataset is provided
RdfStore Benchmarking	RDFS	RDF store	-	-

### Proposed evaluation benchmark (OEB2)

The UOBM [2] is used as a case study for the construction of the proposed benchmark (OEB2) for OWL2 semantics. The reason for selecting UOBM is that it covers most of the OWL constructs in its data schema.

#### Data schema

As a first step, the OEB2 enriches the data schema with the OWL2 constructs. In this regard, three approaches are adopted. First, a survey of the available domain ontologies (e.g. Dbpedia [4], Vehicle [8] and People [omg.org/spec/EDMC-FIBO/FND/AgentsAndPeople/People/]) is performed to check the utilization of OWL2 constructs in the classes and properties of the data schema. Table 3 shows the enriched OWL2 semantics in the OEB2 data schema. Second, WordNet senses are used to add new classes and properties in the data schema [17]. For instance, using WordNet hypernym sense 1 "*has as a graduate*" object property is obtained against "*Has as an Alumnus*" object property under *SameAs* construct. Similarly, Association class is obtained for the already existed Institute class. Third, the formulated object properties pattern queries (details provided later in this section) are executed on the asserted data to obtain the implicit object property characteristics. As a result of queries execution, the OEB2 data schema is enriched with object properties characteristics [18]. For example,

*SELECT distinct*? *p Where {*? *a*? *p*? *b*.? *p rdf*:*type owl*:*ObjectProperty*.? *p rdfs*:*domain*? *k*.? *p rdfs*:*range*? *k filter not EXISTS {*?*a*? *p*? *a}}*

**Table 3:** OWL2 constructs in proposed data schema.

OWL2 constructs	Data schema axioms
All Disjoint Classes	AllDisjointClasses (:ConferencePaper: JournalArticle: TechnicalReport)
Disjoint Union	disjointunion (:ConferencePaper: JournalArticle: TechnicalReport)
Property Chain	SubObjectPropertyOf (ObjectPropertyChain(:subOrganizationOf: subOrganizationOf)
Self Restriction	:Person ObjectHasSelf(: belivesIn)
Reflexivity property	ReflexiveObjectProperty(:likes)
Irreflexivity property	IrreflexiveObjectProperty(:fatherOf)
Asymmetry property	AsymmetricObjectProperty(:fatherOf)
Disjoint object properties	DisjointObjectProperties(:isFriendOf: isOpponentOf)
Disjoint data properties	DisjointDataProperties(: FirstName: LastName)
Keys	HasKey(: Student: hasRegistrationNo)

The above query is written to obtain the likely irreflexive object property characteristic triple pattern from the asserted data of more than 2.3 million triples of the university ontology. The query (mentioned above) returns three object properties *hasSameHomeTownWith*, *isFriendOf* and *subOrganizationOf*. Therefore, these object properties are explicitly expressed as irreflexive property in the data schema.

#### Dataset generator

A number of axioms are supported by OWL2 to describe assertions (i.e. facts about the individuals). These assertions include class, object property and data property assertions. The dataset generator of the OEB2 incorporates all of these OWL2 assertions in its dataset as shown in Table 4.

**Table 4:** OWL2 class assertions in the proposed dataset generator.

OWL2 Assertions	Proposed Dataset generator
SameIndividual	SameIndividual(: AP10: AssistantProfessor10)
DifferentIndividual	DifferentIndividuals(:UnderGraduateStudent1: GraduateStudent22 a:AssociateProfessor13)
ObjectPropertyAssertion	ObjectPropertyAssertion(:fatherOf: AssociateProfessor13: UnderGraduateStudent1)
Negative Data property assertion	Negativedatapropertyassertion(:Assistantprofessor1 telephone number 0323434334)
Negative Object property assertion	NegativeObjectPropertyAssertion(:hasFather: UniveristyGraduate1: AssociateProfessor10)

The OEB2 dataset generator adopts a dynamic approach as compared with the static approach followed by the UOBM. The proposed approach can be used to generate dataset for any given domain. Some important steps involved in the proposed data generation approach are; (i) Selection of classes on the basis of their usage, (ii) Selection of class to serve as root class to represent RDF graph, (iii) Provision to configure number of instances to be generated against the selected classes, (iv) Selection of properties and making of assertion statements.

In the first step, we take the mean value of each class as a measure to find out to which degree a class is used as subclass of relations, restriction, domain and range in the data schema. Later, classes are ranked according to their mean value and only top quadrant is selected. In our case 32 classes out of 130 classes are chosen. The selected classes are then mapped to their original hierarchy to form clusters for determination of the candidate classes that are suitable for serving as a root class. As a result, among the five formulated clusters (organization, person, publication, course and research) two main clusters are chosen, led by Person and Organization class respectively. In the second step, a root class is dynamically selected (i.e. Organization class) from the main clusters on the basis of superiority of domain and range associations. In the third step, the configuration for generating instances for each selected class is based on the depth of the class, its mean value and a random number (between 10 to 100). In the last step, properties are selected whose range and domain belong to the selected classes. These selected properties contain both OWL and OWL2 property characteristics while assertion statements generation follows the UOBM [2].

#### Queries set

The proposed benchmark queries are divided into simple, complex and object properties characteristics pattern queries (OPQ). Simple Queries (SQ) are about checking OWL2 constructs from structural point of view such as, distinct property characteristics, domain, range and disjoint union. Complex Queries (CQ) include bushy patterns, long chains, large size and irregular pattern queries. The OPQs are used to retrieve the object property characteristics patterns (e.g. Transitive, Inverse functional, Asymmetric, Functional). The details of the queries are provided in the technical report (https://github.com/azeemabbas/oeb2Benchmark).

## Evaluation of the OEB2 benchmark

**Fig 1:**
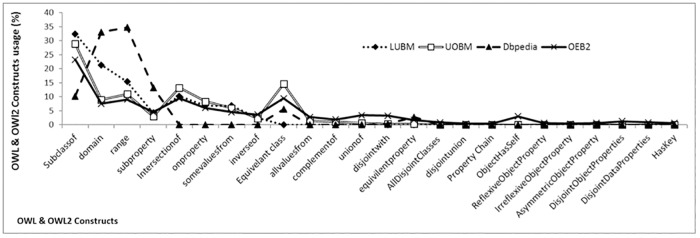
Comparison of OWL & OWL2 constructs in the OEB2 and existing benchmarks data schemas.

**Fig 2:**
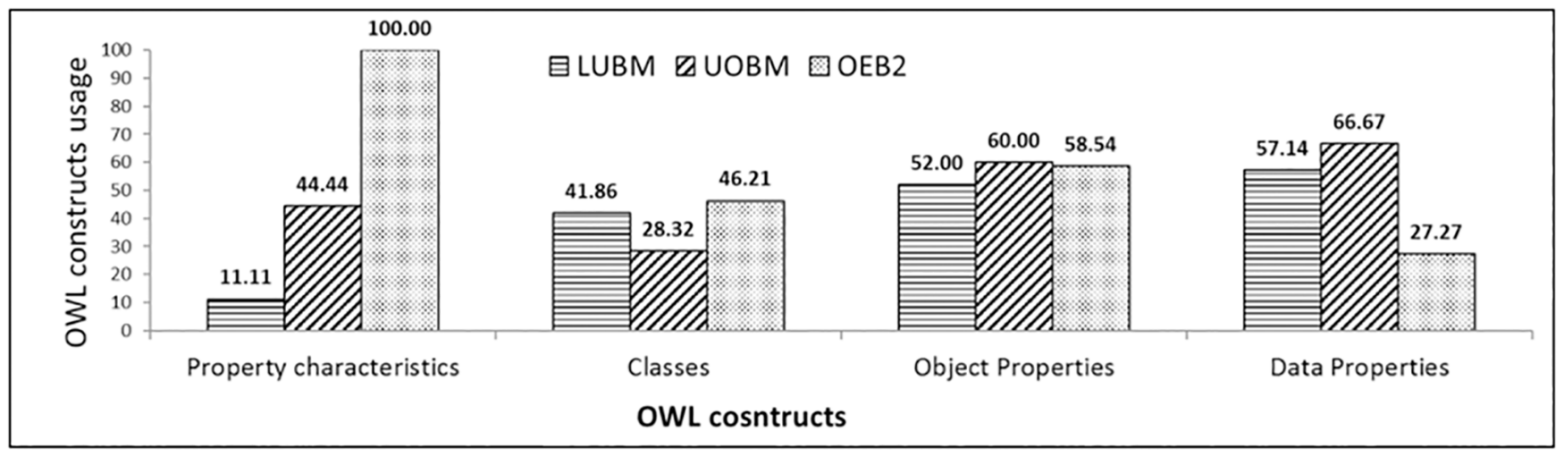
Ontology constructs usage by the benchmark dataset generators.

**Fig 3:**
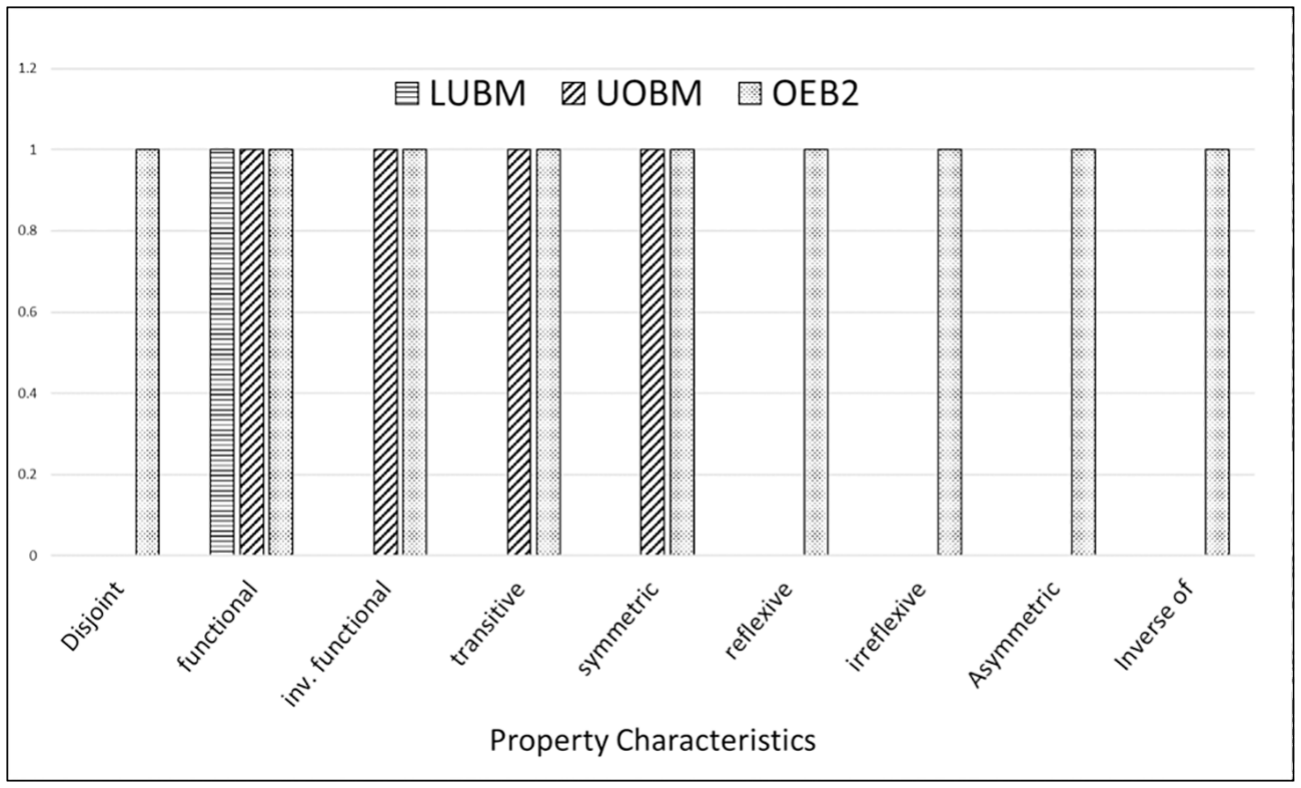
Property characteristics usage by the benchmark dataset generators.

The evaluation of the proposed benchmark (OEB2) is carried out by comparing the data schemas and dataset generators of the existing benchmarks with OEB2’s data schema and dataset generator for OWL2 semantics. The OEB2 addresses all of the OWL and OWL2 constructs in its data schema as compared to the existing benchmarks (Fig 1). In the Fig 1, the x-axis represents the OWL/OWL2 constructs and y-axis describes their usage (scaled logarithmic). The Fig 1 shows that Dbpedia [19] has high use of SubClassOf relation as compared to the other benchmarks. This concludes that Dbpdia is more inclined toward hierarchal organization as compared with association centric. While, OEB2 uses all of the ontology constructs even if their usage is at a minimum. This shows that the data schema of OEB2 is balanced between concept organization and property associations. Fig 2 shows the comparison of ontology constructs usage in the dataset generators. OEB2 provides hundred percent use of the object property characteristics as compare to LUBM and UOBM. The reason is shown in Fig 3, which states that OEB2 uses all the nine (09) object property characteristics while LUBM and UOBM uses only one (01) and (04) object property characteristics respectively. The usage of Classes and Object properties in OEB2 is similar to the other benchmarks (Fig 2). Moreover, OEB2 dynamically selects Classes and Properties as compared with the static approach of existing benchmarks. For generation of instances, OEB2 adopts a concise approach in contrast to the exhaustive approach followed by existing benchmarks. For example, isFriendOf is a reflexive property; then only one statement would express both directional relationship between two distinct individuals as followed by OEB2 (i.e. A *isFriendOf* B inherently covers B *isFriendOf* A). The OEB2 benchmark queries are evaluated by comparing the coverage of ontology constructs in OEB2 and existing benchmark queries. The proposed queries have a higher coverage of ontology constructs as compared to other existing benchmarks (see technical report). The reason is that, most of the OEB2 queries are generic in nature. It means a query does not look for a specific class instance, rather instances are retrieved against the object property characteristics or a queried pattern e.g. bushy patterns and long chain queries. Moreover, the classification of OEB2 queries supports evaluation of different domains ranging from simple to high complexity. This will help the researchers to use the structural complexities of the KBS.

## Results and discussion

In this section, well known KBSs are evaluated using OEB2 benchmark for performance metrics. Three synthetic data sets of size 24K, 240K and 2400K triples are generated from OEB2 data schema for the said evaluation. The smallest dataset comprises of more than 24 thousand triples and the largest dataset (2400K) comprises of more than 2.5 million triples. The semantic tools used for conducting the experimentation are Jena API, OpenRDFWorkbench, Protégé, MySQL and SQL Server on Intel^®^ Core i5-4200M CPU @ 2.5 GHz with 6 GB RAM. The KBS used for evaluation includes Sesame (in memory), Sesame (DB), Jena SDB, Blazegraph graph based system, RDF Native storage system, OWL2ToRDB and OntRel database system. Similar to UOBM [2], load time and response time are used as performance metrics. Load time refers to the time required for loading a dataset into memory or persistence storage. The response time is the time required for issuing a query, obtaining and traversing the results sequentially. The dataset generated by OEB2 reflects the true behaviour for load time of an underlying KBS because of its high data schema complexity, larger coverage of the ontology constructs (Figs 1 and 2) and dynamic generation of dataset (Section 3).

**Fig 4:**
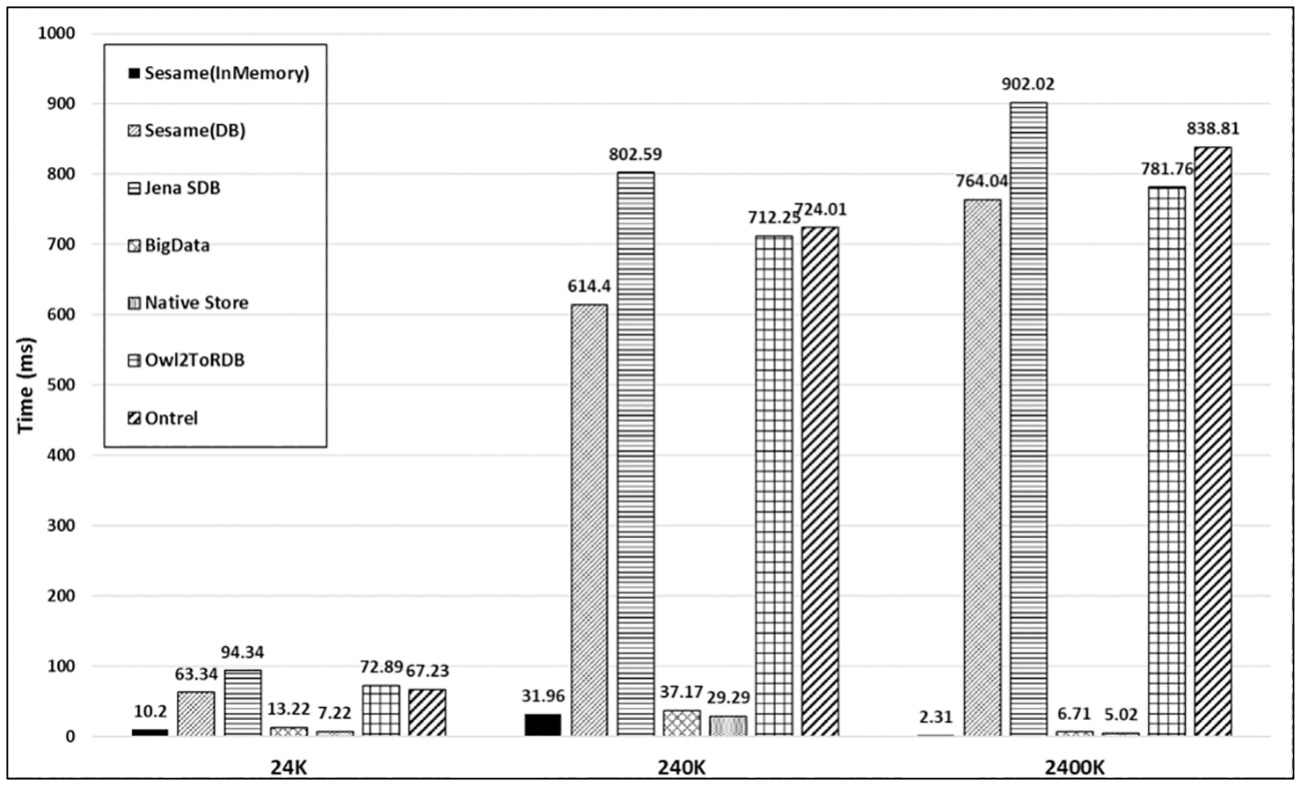
Load time of different ontology storage systems on different size datasets.

Fig 4 shows the load time of the KBSs. Sesame (in memory), Native Store and Blazegraph is less than 20 seconds to load 24K triples as compared with the database storage systems (Sesame(DB), Jena SDB, OWL2toRDB and OntRel). For larger datasets (240K and 2400K), the non-database systems perform better than other systems. The values of the Sesame (in memory), Blazegraph and Native Store are too small to plot on the figure. Overall, Sesame (in memory) performs faster in loading the datasets.

**Fig 5:**
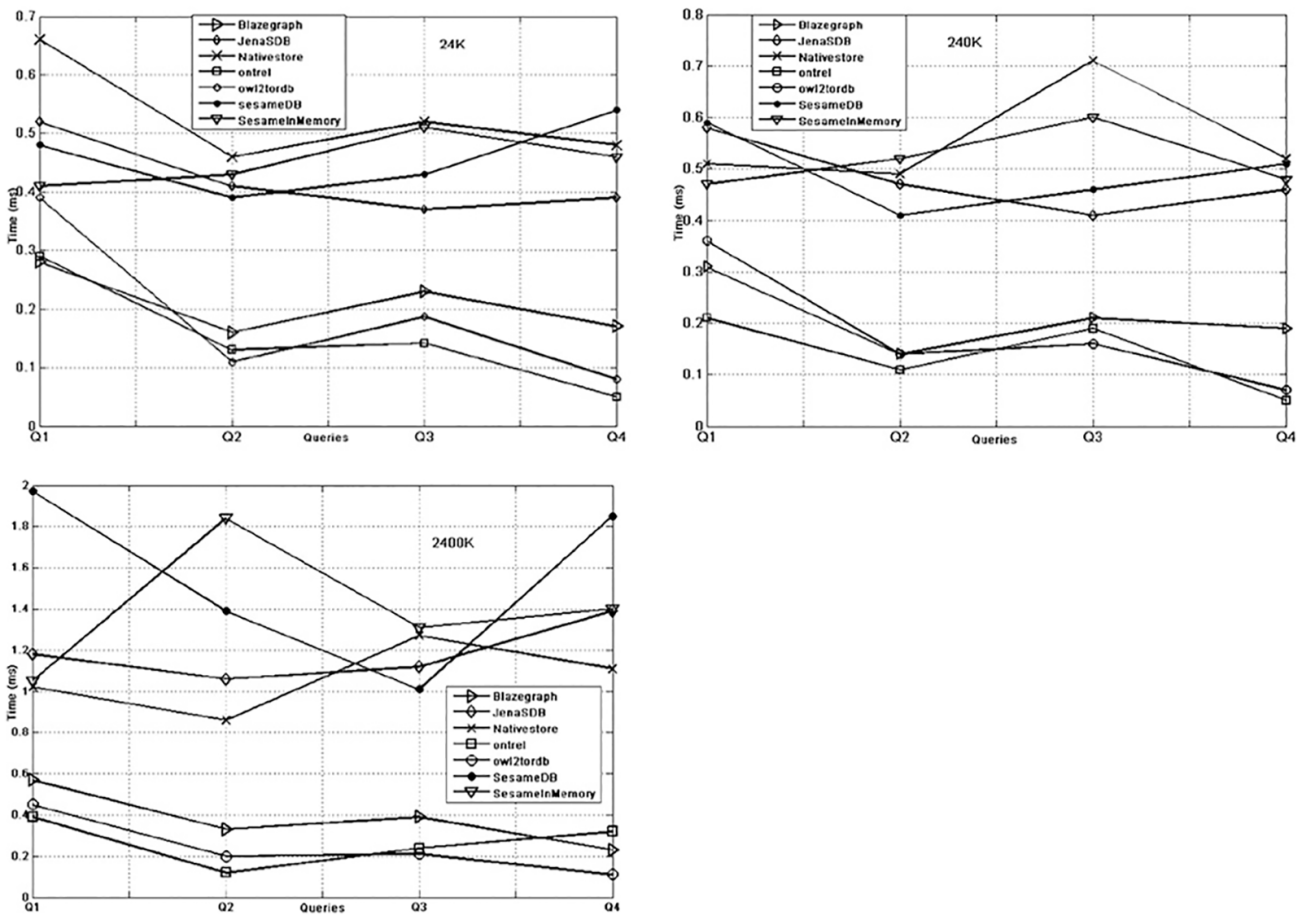
Simple query response time of KBS on 24K (a), 240K (b) and 2400K (c) triples.

**Fig 6:**
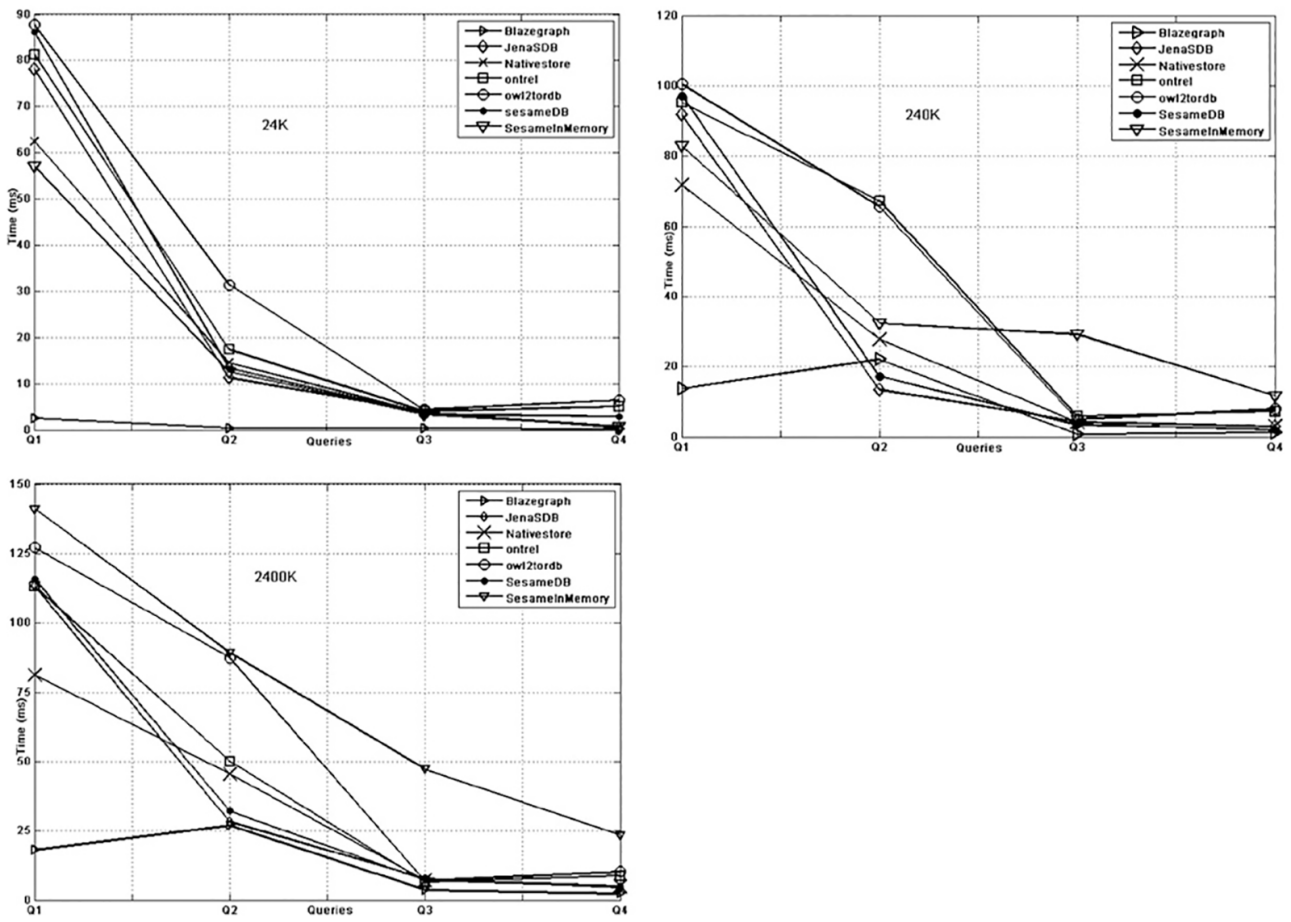
Complex query response time of KBS on 24K (a), 240K (b) and 2400K (c) triples.

The query response time of different KBS on simple queries is very small as shown in Fig 5 and the prime reasons include nature of the queries and the limited result set. The behaviour of KBS against simple queries remains the same for the datasets in different sizes. However, the performance of Blazegraph, Owl2ToRDB and OntRel is better than other systems. For complex queries the behaviour of KBSs varies on different size datasets. Fig 6(a) shows that except Blazegraph, the response time of different queries remain the same. In Fig 6(b), the database systems and in-memory system have high response time on bushy pattern and long chain queries (Q1,Q2) as compared to Blazegraph and Native store. For data size 2400K as seen in Fig 6(c), the gap widens and only Blazegraph shows better performance on bushy pattern and long chain queries (Q1, Q2). While, for the queries (Q3,Q4) the behaviour of Sesame DB, Jena SDB, Ontrel and Owl2ToRDB is similar to Blazegraph and Native store systems.

**Fig 7:**
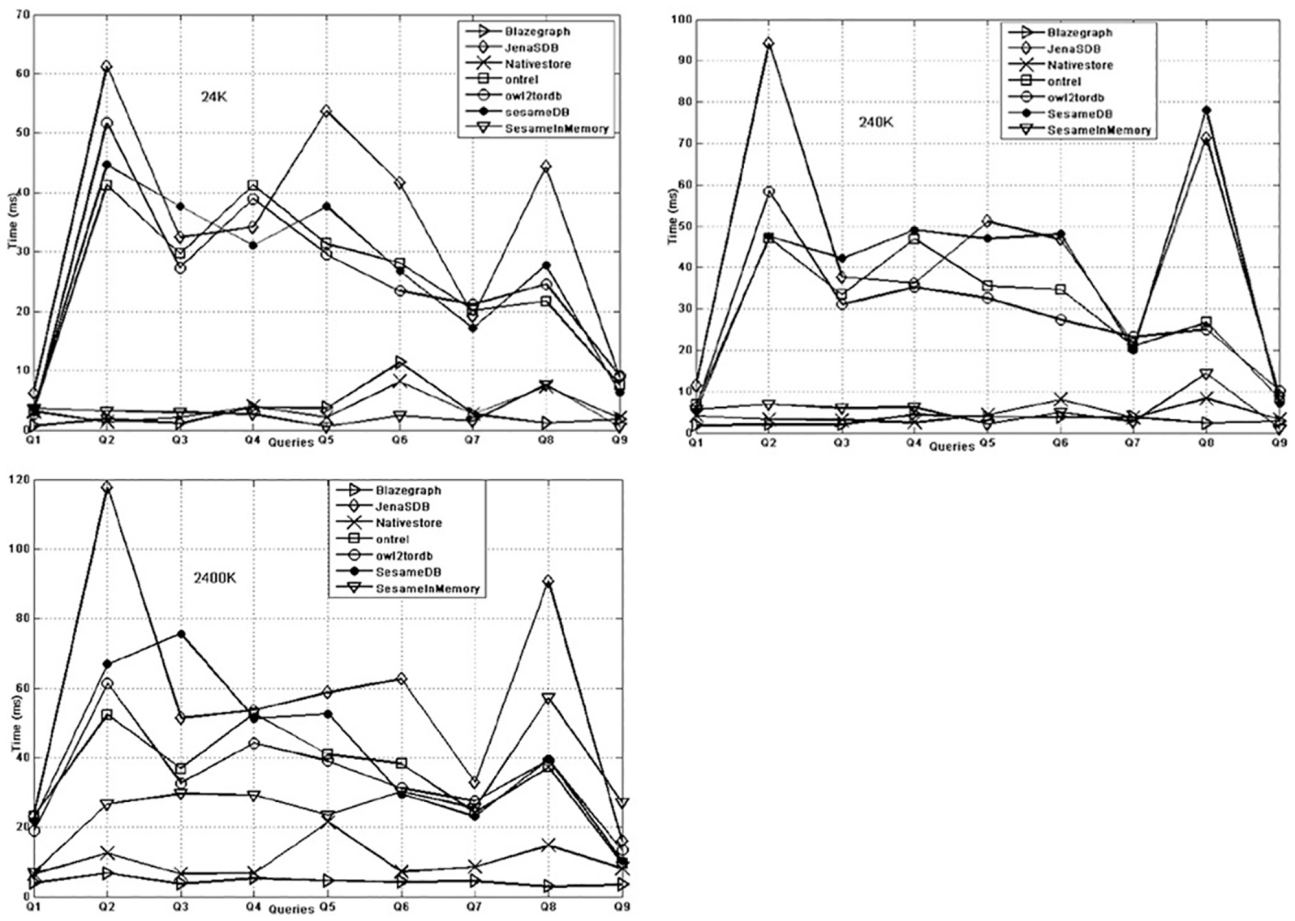
Property pattern query response time of KBS on 24K (a), 240K (b) and 2400K (c).

In the (third type of queries) property characteristics pattern queries, the query response time is shown in Fig 7, which states that on dataset size 24k, the behaviour of in-memory, graph and native systems is far better than the database systems (Ontrel and Owl2ToRDB). The gap between memory and database systems is reduced on 240K triples dataset. In Fig 7(c), the gap is further reduced and gives overall trend that as the data size increases the performance of memory and native store is degraded as compare with the database systems.

As a summary of the results, Blazegraph, Owl2ToRDB and Ontrel are efficient in searching for distinct properties, domain and range, subject and predicates of triples for simple queries. Moreover, the performance of Owl2TRDB and Ontrel is better over large size datasets. The reason for this efficiency is the usage of meta tables for ontology constructs. Therefore, these KBSs are suitable choice for domains requiring simple queries i.e. Agriculture domain. For complex queries, Blazegraph out performs in bushy pattern queries, while Ontrel better performs on long chain queries over large size dataset. Therefore, theses KBSs can be a better choice in domains having long chains i.e. medical and scientific domains. For high selectivity or the large result set, all the KBS exhibits better behaviour over large datasets except the Sesame (In memory). For irregular pattern queries Blazegraph shows stability as the dataset size grows. This shows that Blazegraph is a suitable choice for domains where irregular pattern queries are frequently used i.e. gene ontology. For OPQs, the performance of Blazegraph, Sesame (in memory) and RDF native store (Sesame) performs well as compared with others. In contrast, the performance of relational database KBS (i.e. Sesame DB, Jena SDB, Ontrel and OWL2TRDB) for OPQs is very poor over large size datasets. On the basis of the results, it is concluded that memory systems are suitable for the domains where queries generate relatively small result size. Over all Blazegraph has shown stable performance except slight performance degradation on bushy pattern (Q1) and irregular pattern (Q2) queries.

## Conclusion

The present work contributes the research domain by addressing the question "which KBS is more suitable for any specific domains using OWL2 semantics?” The results reported by the proposed benchmark (OEB2) clearly shows the suitability of KBS for domain specific needs. We have evaluated and compared seven different KBSs belonging to memory, persistence storage, relational database and graph based. The evaluation results of the proposed benchmark identify the strengths and weaknesses of the KBSs. The Future work on OWL2 benchmarking for the evaluation of KBS can adopt the generic dataset generator of OEB2 for the evaluation of different domains to get more realistic and quantifiable results.

## Abbreviations

API: Application Program Interface
BSBM: Berlin Sparql Benchmark
CQ: Complex Queries
CRUD: Create Read Update and Delete
ENR: Edge Node Ratio
KBS: Knowledge Base System
LBBM: Lehigh Bibtex Benchmark
LUBM: Lehigh University Benchmark
MIS: Management Information System
OEB2: Ontology Evalu0ative Benchmark
OPQ: Object Properties Characteristics Pattern Queries
OWL: Web ontology language
OWL2: Web ontology Language Version 2
RAM: Random Access Memory
RDF: Resource Description Framework
RDFS: Resource Description Framework Schema
SQ: Simple Queries
UOBM: University Ontology Benchmark
